# The long road after breast cancer: survivorship issues and long-term treatment effects in young women

**DOI:** 10.3389/fonc.2026.1840152

**Published:** 2026-06-17

**Authors:** Martina Pagliuca, Roberto Buonaiuto, Annarita Verrazzo, Sara Parola, Margherita Tafuro, Claudia Calderaio, Claudia Martinelli, Rossana Di Rienzo, Miriam Pirolo, Christian Zurlo, Ornella Tinelli, Roberta Valente, Luisa Cirillo, Martina Autiero, Maria Luisa D’Aulisio Garigliota, Raffaella Di Monda, Carmine De Angelis, Michelino De Laurentiis

**Affiliations:** 1 Clinical and Translational Oncology, Scuola Superiore Meridionale, Naples, Italy; 2 Université Paris-Saclay, UVSQ, Gustave Roussy, Inserm, CESP, Villejuif, France; 3 Department of Breast and Thoracic Oncology, IRCCS Istituto Nazionale Tumori “Fondazione G. Pascale”, Naples, Italy; 4 Université Libre de Bruxelles (ULB), Hôpital Universitaire de Bruxelles (H.U.B), Institut Jules Bordet, Bruxelles, Belgium

**Keywords:** breast cancer, long - term effect, psychological issues, survivorship care, young survivors

## Abstract

Advances in early detection and treatment have significantly improved survival rates in young women with breast cancer, leading to a growing population of long-term survivors. However, survivorship in this population is characterized by a unique and complex set of challenges that extend well beyond the completion of active treatment. This review aims to provide a comprehensive overview of the long-term physical, psychological, reproductive, and social effects of breast cancer and its treatment in young survivors, highlighting the need for tailored, age-specific survivorship care. Young breast cancer survivors are particularly vulnerable to treatment-related sequelae due to the interaction between therapy-related toxicities and critical life stages, including family planning, career development, and relationship building. Common long-term physical effects include bone density loss associated with premature ovarian insufficiency, cognitive dysfunction, and persistent cancer-related fatigue. In addition, endocrine therapies and treatment-induced menopause contribute to vasomotor symptoms (VMS), sleep disturbances, weight gain, and sexual dysfunction, all of which can significantly impair quality of life. Psychological distress is highly prevalent in this population, with elevated rates of anxiety, depression, post-traumatic stress, and fear of cancer recurrence. Fertility and reproductive health concerns represent a central issue, encompassing fertility preservation, pregnancy after breast cancer, and the safety of interrupting endocrine therapy. Social challenges, including difficulties with return to work, financial toxicity, and disruptions in intimate relationships, further compound the survivorship burden. Despite increasing recognition of these issues, many remain underdiagnosed and undertreated. A multidisciplinary, patient-centered approach is essential, integrating oncologic care with primary care, reproductive endocrinology, mental health services, and supportive care interventions. Early counseling, routine screening, and proactive management of long-term effects are key components of optimal survivorship care. By synthesizing current evidence, this review underscores the importance of developing comprehensive survivorship strategies tailored to the specific needs of young breast cancer survivors, with the ultimate goal of improving long-term outcomes and enabling patients not only to survive but to thrive after breast cancer.

## Introduction

Over the past few decades, advances in breast cancer treatment and early detection have led to a significant increase in survival rates, with more young women living years beyond diagnosis. While this progress represents a remarkable achievement in modern medicine, it also highlights the multiple, long-term challenges that young breast cancer survivors face after the initial treatment phase (1). These challenges may be particularly pronounced in younger patients, who are often navigating critical life stages such as family planning, career establishment, intimate relationships, and identity development, all of which can be profoundly disrupted by a cancer diagnosis and its treatment. Cancer survivorship has evolved into a distinct and complex journey, encompassing several phases, from early recovery to long-term adjustment. These stages often involve ongoing physical side effects, emotional distress, and social reintegration issues that affect family life, work, body image, and personal relationships (2). Young survivors frequently face a combination of chronic health problems and psychological challenges, including heightened anxiety about cancer recurrence, concerns about premature menopause and infertility, and disruptions to sexual health and functioning, all of which can complicate their return to normal routines and undermine mental well-being (3).

The growing population of young breast cancer survivors has raised awareness of the unique, ongoing needs of this group, and the field of survivorship care has evolved to address them. Survivors often experience persistent physical symptoms such as fatigue, chronic pain, and organ-related side effects, as well as musculoskeletal symptoms and vasomotor effects from endocrine therapy (ET). The importance of survivorship care becomes even more evident during extended ET, which may be prolonged for up to a decade and can lead to cumulative physical symptoms and psychological distress that ultimately compromise both quality of life and treatment adherence. Psychological challenges, including depression, anxiety, fear of recurrence, and grief over lost fertility or altered reproductive plans, can have a significant impact on quality of life, making reintegration into society, family roles, and work environments a delicate process (4). Chemotherapy-related amenorrhea, in particular, has been associated with worse insomnia and impaired sexual functioning, adding to the burden of survivorship in this age group (5). In addition, survivors often experience a heightened sense of vulnerability as they transition away from frequent contact with oncology teams, which can leave them feeling disconnected from important support systems at a time when they are also confronting decisions about fertility preservation, pregnancy timing, and the safety of interrupting ET. A comprehensive, patient-centered approach to survivorship care is therefore essential for young breast cancer survivors, with an emphasis on holistic health, emotional support, reproductive counseling, and proactive management of ongoing treatment effects (2).

**Figure 1:**
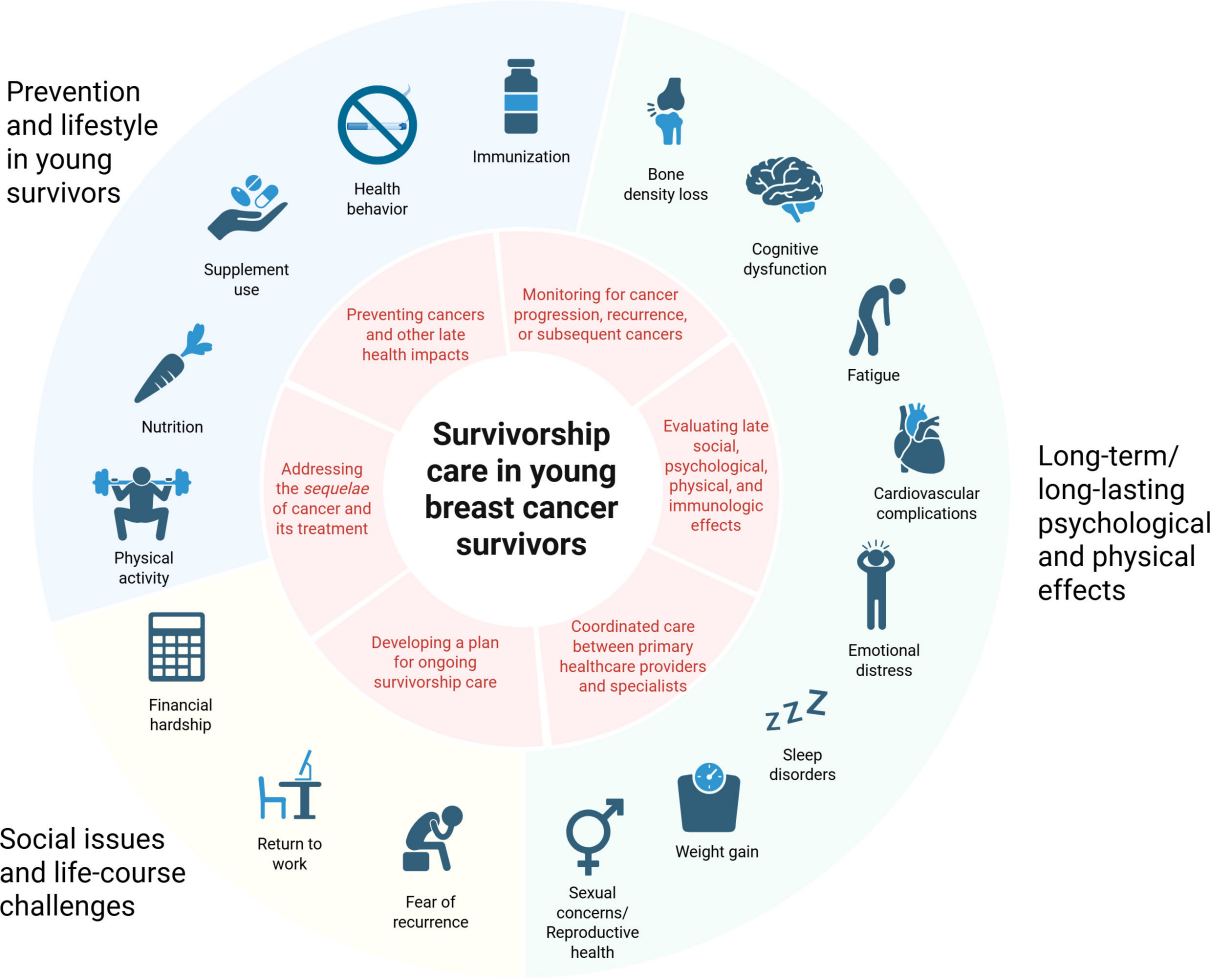
Multidimensional long-term effects and survivorship care in young breast cancer survivors. Created with BioRender.com.

To meet these diverse needs, experts advocate for survivorship care plans tailored to young women that include ongoing health monitoring, preventive measures, fertility and sexual health support, and strategies to address long-term physical and psychological effects (4). Coordinated care that includes oncologists, primary care providers, reproductive endocrinologists, and mental health professionals is essential to improving survivors’ quality of life. Despite the growing recognition of these needs, long-term symptoms and survivorship-related challenges in young breast cancer patients are still not routinely or systematically assessed in daily clinical practice. Structured survivorship programs and dedicated multidisciplinary services remain limited in many institutions, and management is often dependent on individual specialists rather than coordinated care pathways. Furthermore, survivorship care is not consistently incorporated into oncology training programs, contributing to substantial variability in awareness and clinical management across different healthcare settings. As the field continues to grow, the importance of integrated survivorship programs and policies that prioritize the well-being of this young and diverse patient population is being increasingly emphasized. For this narrative review, a literature search was conducted in PubMed and Embase, together with consultation of major international oncology and survivorship guidelines, to identify studies addressing survivorship issues and long-term treatment effects in young breast cancer survivors. Relevant English-language publications, including clinical trials, observational studies, meta-analyses, and guideline recommendations, were selected based on their relevance to the topics covered. This review explores many critical long-term and long-lasting effects of breast cancer treatment in young survivors and highlights the need for further research to improve survivorship care for those who live beyond breast cancer. The key domains of survivorship care and long-term effects in young breast cancer survivors are summarized in **Figure 1** and **Table 1**.

**Table 1:** Survivorship issues and long-term treatment effects in young breast cancer survivors.

Symptom/problem	Associated / overlapping symptoms	Underlying mechanisms	Current management recommendations
Bone density loss / Osteoporosis	• Fracture risk • Joint pain • Muscle weakness • Falls • POI	• Chemotherapy-induced POI → rapid estrogen loss • AIs + GnRH agonists → ↑ osteoclast activity (up to 7.7% annual BMD loss) • Tamoxifen → bone loss in premenopausal women • >80% of BC patients experience some degree of bone loss	**Screening (LoE Category 2A):** • Baseline DEXA scan; repeat periodically in patients on AIs or with treatment-induced ovarian failure • Fracture risk assessment (FRAX) **Non-pharmacological:** • Calcium 1,200 mg/day total intake • Vitamin D3 600–1,000 IU/day • Weight-bearing exercise (LoE Category 2A) • Smoking cessation; limit alcohol (LoE Category 2A) **Pharmacological:** • Bisphosphonates (zoledronic acid, alendronate) for high-risk patients (LoE Category 2A) • Denosumab as alternative antiresorptive agent (LoE Category 2A)
Cancer-associated cognitive dysfunction	• Fatigue • Depression / anxiety • Sleep disturbance • Pain • Attention, memory, executive function deficits	• Chemotherapy neurotoxicity: white matter damage, altered brain activity patterns • Treatment-induced POI and GnRH agonist therapy • Elevated pro-inflammatory cytokines • HPA-axis disruption • Symptom clustering with fatigue, mood disorders	**General strategies:** • Memory aids, organizational tools, reminders • Optimize co-occurring contributors: depression, sleep, fatigue, pain **First-line (LoE Category 2A):** • Neuropsychological evaluation and testing • Cognitive rehabilitation (occupational therapy, speech therapy) • Psychotherapy • Routine physical activity • Mindfulness-based stress reduction; cognitive training **Second-line (LoE Category 2A):** • Consider methylphenidate, modafinil, or donepezil (limited evidence)
Cancer-related fatigue	• Pain • Depression / anxiety • Anemia • Sleep disturbance • Cognitive dysfunction • Weight gain	• Inflammation / immune dysregulation (↑ pro-inflammatory cytokines) • Hormonal dysregulation (treatment-induced POI, HPA-axis disruption) • Anemia • Sedentary lifestyle; higher BMI • Psychosocial burden amplified in young women	**Contributing factors:** • Treat pain, emotional distress, anemia, sleep disturbance, nutritional deficits, comorbidities **Physical activity (LoE Category 1):** • Aerobic exercise + muscle strength training (most evidence) • Home-based walking, yoga, aquatic exercise **Psychosocial (LoE Category 1):** • CBT / behavioral therapy (most effective per network meta-analysis) • Mindfulness-based stress reduction (MBSR) • Psycho-educational therapies • Supportive expressive therapies • Massage therapy **Other non-pharmacological:** • CBT for insomnia (CBT-I) (LoE Category 1) • Acupuncture (LoE Category 2A) • Bright white light therapy (LoE Category 2A) • Nutrition consultation **Pharmacological (investigational):** • Methylphenidate: only after ruling out other causes and if non-pharmacological interventions fail
Emotional distress	• Sleep disturbance • Fatigue • Cognitive dysfunction • Social withdrawal • Suicidal ideation (10% pooled prevalence) • Altered body image • Fertility concerns	• Abrupt life-stage disruption (career, family planning, relationships) • Fear of cancer recurrence (FCR; 70–84% in young adults) • Treatment-induced body changes and hormonal shifts • Chronification of trauma response • Low social support; financial strain; dependent children	**Screening:** • Routine screening with validated tools: PHQ-9, GAD-7, PC-PTSD-5, Distress Thermometer • Re-screen at transitions, surveillance appointments, major life events **Non-pharmacological:** • CBT and behavioral therapies (preferred first-line; LoE Category 1) • Psychotherapy / psycho-oncology referral (LoE Category 2A) • Mindfulness-based stress reduction (LoE Category 2A) • Supportive expressive group therapies (LoE Category 2A) • Fear of recurrence-specific interventions (LoE Category 2A) **Pharmacological:** • SSRIs / SNRIs as first-line antidepressants (LoE Category 2A; caution with tamoxifen for some SSRIs) • Psychiatric referral for PTSD, severe anxiety, suicidal ideation
Sleep disorders	• VMS (hot flashes/night sweats) • Depression / anxiety • Fatigue • Cognitive dysfunction • Pain	• Treatment-induced premature menopause → hormonal disruption of sleep-wake pathways • VMS directly fragmenting sleep • Psychological stress, rumination • Medication side effects (ET, corticosteroids, antiemetics) • Maladaptive sleep behaviors (irregular schedules, daytime napping)	**Screening:** • Regular assessment of contributing factors: VMS, medications, pain, fatigue, emotional distress **First-line (LoE Category 2A):** • CBT for Insomnia (CBT-I): preferred - includes stimulus control, sleep restriction, sleep hygiene; shown effective via internet delivery (large effect size, d=1.17) • Treat associated VMS **Non-pharmacological (LoE Category 2A):** • Sleep hygiene • Mindfulness-based stress reduction programs • Physical activity • Acupuncture **Pharmacological (LoE Category 2A):** • Benzodiazepines (e.g., lorazepam): short-term use when non-pharmacological insufficient • Referral to sleep specialist for refractory or chronic insomnia
Weight gain	• Fatigue • Cardiovascular disease risk • Insulin resistance / diabetes • Dyslipidemia • Bodyimage concerns • Recurrence risk	• Chemotherapy-induced POI → estrogenic loss → metabolic dysregulation • AIs → ↑ fat mass, ↓ lean mass (sarcopenic-obesity phenotype) • Tamoxifen • Reduced physical activity during treatment • Psychosocial eating behaviors	**Goal:** • Achieve BMI 18.5–24.9 kg/m² **First-line multimodal interventions (LoE Category 2A):** • Regular physical exercise (most evidence: aerobic + strength training) • Dietary counseling / caloric management • CBT / behavioral modification • In-person or remote/digital programs: both effective and superior to standard medical care **Additional (LoE Category 2A):** • Weight loss of ≤2 lb/week recommended • Pharmacological treatment of metabolic comorbidities (dyslipidemia, insulin resistance) as needed • No pharmacological intervention proven specifically effective for ET-related weight gain
VMS	• Sleep disturbance • Depression / anxiety • Fatigue • Sexual dysfunction • ET non-adherence	• Estrogen deprivation from chemotherapy, ovarian suppression, AIs, tamoxifen • Altered hypothalamic thermoregulatory center function • Neurotransmitter dysregulation (serotonin, noradrenaline, neurokinin-3 pathway) • Abrupt estrogen loss in young women → more pronounced than natural menopause	**Pharmacological - non-hormonal (first-line) (LoE Category 2A):** • Venlafaxine (SNRI): 75 mg/day; safe with tamoxifen • Desvenlafaxine, escitalopram, citalopram (SSRIs/SNRIs with mild CYP2D6 interaction) • Gabapentin (anticonvulsant): 900 mg/day; similar efficacy to venlafaxine • Pregabalin: 150–300 mg/day • Oxybutynin: 5–10 mg/day (anticholinergic) • Elinzanetant (NK1/NK3 antagonist, preferred): 120 mg/night - studied in patients on ET for HR+ BC • Fezolinetant (NK3 antagonist): 45 mg/day; >50% VMS reduction; BC patients excluded from original trials **Non-pharmacological (LoE Category 2A):** • CBT • Acupuncture • Yoga / mindfulness-based interventions • Physical activity / exercise • Stellate ganglion block (selected cases) • Hypnosis • Weight loss if overweight/obese • Cool pad pillow (for sleep-related symptoms)
Sexual dysfunction / GSM	• Vasomotor symptoms • Sleep disturbance • Depression / anxiety • Altered body image • Relationship difficulties • Pain (dyspareunia)	• Estrogen deprivation → vaginal/urogenital epithelium thinning, dryness • AIs + ovarian suppression: highest GSM rates • Psychological: anxiety, depression, body image changes, fertility concerns • Surgical changes (mastectomy, reconstruction) impacting body image	**First-line - non-hormonal:** • Vaginal lubricants and moisturizers (water-based, silicone-based, hyaluronic acid): offered to all survivors with vaginal symptoms (LoE Category 2B) • Pelvic floor physiotherapy (LoE Category 2A) • Vaginal dilators (LoE Category 2A) • Topical 4% aqueous lidocaine compresses for dyspareunia (LoE Category 2A) **Second-line(LoE Category 2A):** • Vitamin D/E vaginal suppositories • Vaginal CO2 or erbium laser therapy (awaiting robust RCT data) • Intravaginal oxygen + hyaluronic acid (emerging approach) **Hormonal (use with caution, LoE Category 2B):** • Low-dose vaginal estrogen (rings/suppositories preferred over cream): may be considered for severe refractory symptoms after risk discussion; generally not recommended with AIs **Psychosocial (LoE Category 2A):** • CBT: improves sexual function, desire, arousal, vaginal lubrication, body image, sexual distress • Couples counseling; sex therapy • Psychosexual intervention programs for young women on ovarian suppression **Screening:** • Screen for sexual health concerns at regular intervals; refer to sexual health specialist as needed
Cardiovascular complications	• Weight gain / obesity • Dyslipidemia • Insulin resistance / diabetes • Hypertension • Bone density loss • Fatigue	• Chemotherapy-induced POI → abrupt estrogen deprivation decades early • Anthracyclines → endothelial injury, arterial stiffness, vascular aging phenotype • AIs → ↑ visceral adiposity, ↓ lean mass, atherogenic lipid profile (↑ LDL, ↑ TG, ↓ HDL) • POI → impaired endothelial NO production, oxidative stress, insulin resistance, low-grade inflammation • Sarcopenic-obesity phenotype → cardiometabolic risk	**Screening and surveillance (LoE Category 2A):** • Regular cardiovascular risk factor assessment: BP, lipids, weight, BMI, metabolic syndrome screening • Echocardiography for anthracycline-exposed patients as clinically indicated **Lifestyle interventions:** • Weight management targeting BMI 18.5–24.9 kg/m² • Regular aerobic and resistance exercise (↓ cholesterol, TG, insulin resistance) • Heart-healthy diet; smoking cessation; limit alcohol **Pharmacological (as per primary care / cardiologist):** • Treat dyslipidemia, hypertension, insulin resistance per standard guidelines • Referral to cardiologist for high-risk patients or pre-existing cardiac disease
Fertility and reproductive health concerns	• Premature ovarian insufficiency • VMS • Sexual dysfunction • Anxiety / depression • FCR • Bone density loss	• Chemotherapy-induced gonadotoxicity • Amenorrhea ≠ infertility; menses resumption does not guarantee fertility	**Counseling at diagnosis (LoE Category 2A):** • All premenopausal patients informed of treatment impact on fertility and asked about reproductive goals • Timely referral to fertility specialist when appropriate **Fertility preservation (LoE Category 2A):** • Oocyte and embryo cryopreservation: established standard strategies • Ovarian tissue cryopreservation: in selected cases • GnRH agonists during chemotherapy: adjunct to reduce ovarian failure risk (LoE Category 2A) **Contraception (LoE Category 2A):** • Non-hormonal methods preferred; hormone-based contraception generally discouraged **Pregnancy after breast cancer:** • Generally safe; no demonstrated increase in recurrence risk or adverse fetal outcomes • Temporary interruption of ET for pregnancy (POSITIVE trial): safe option in selected patients; 72% achieved pregnancy, majority resumed ET; long-term data still emerging **Access and equity:** • Address financial barriers to fertility preservation; disparities in access must be recognized
Psychosocial and social issues	• Emotional distress • Fatigue • Cognitive dysfunction • Lymphedema • FCR • Altered body image • Fertility concerns	• Treatment occurs during critical life-stage • Treatment-related adverse events (fatigue, lymphedema, cognitive dysfunction) impair work performance • Financial burden: direct treatment costs + indirect (lost income, reduced work capacity)	**Screening (LoE Category 2A):** • Annual psychosocial assessment; financial toxicity screening • Screen for work, school, and relationship challenges using validated tools (e.g., Distress Thermometer problem list) **Return to work:** • Occupational therapy / vocational rehabilitation referral • Workplace accommodations (gradual return, modified duties) • Treat contributing factors: fatigue, lymphedema, cognitive dysfunction, depression **Financial toxicity:** • Social work referral for financial counseling and resource navigation • Insurance and disability benefits guidance **Digital and remote interventions:** • eHealth tools, mobile apps, and self-monitoring devices: promising for young survivors more receptive to technology-based approaches

Sources: NCCN Survivorship Guidelines v2.2026.

AIs, aromatase inhibitors; BC, breast cancer; BMD, bone mineral density; CBT, cognitive behavioural therapy; CBT-I, CBT for insomnia; DEXA, dual-energy X-ray absorptiometry; ET, endocrine therapy; FCR, fear of cancer recurrence; GnRH, gonadotropin-releasing hormone; GSM, genitourinary syndrome of menopause; HPA, hypothalamic-pituitary-adrenal; HR+, hormone receptor positive; LoE, Level of evidence; MBSR, mindfulness-based stress reduction; MHT, menopausal hormone therapy; NK3, neurokinin-3; POI, premature ovarian insufficiency; RCT, randomised controlled trial; SNRI, serotonin-noradrenaline reuptake inhibitor; SSRI, selective serotonin reuptake inhibitor; VMS, vasomotor symptoms.

## Potential long-term effects of cancer therapy

### Bone density loss

Breast cancer treatment can significantly affect bone health, leading to an increased risk of osteoporosis characterized by low bone density and structural deterioration that predispose to fractures. Up to 80% of breast cancer patients experience bone loss, with the rate and magnitude of therapy-related bone loss significantly exceeding normal age-related loss (4). This issue is particularly relevant in young breast cancer survivors, in whom chemotherapy-induced premature ovarian insufficiency (POI) causes rapid and profound bone loss with lumbar spine bone mineral density (BMD) decreasing by as much as 4% within the first 6 months and continuing to decline thereafter (6). More than half of breast cancer survivors under 40 have severely reduced ovarian reserve after chemotherapy, increasing the risk of POI and its skeletal consequences. Additionally, young patients under 45 with newly diagnosed breast cancer face a nearly 9-fold higher risk of abnormal bone mass compared to age-matched controls (7). ETs further compound the problem: aromatase inhibitors combined with GnRH agonists in premenopausal patients can cause annual lumbar spine BMD losses of up to 7.7%, while tamoxifen, unlike its bone-protective effect in postmenopausal women, has been shown to cause significant bone loss in premenopausal patients (8, 9). To manage these risks, guidelines recommend baseline and periodic DEXA screening for patients receiving aromatase inhibitors or experiencing treatment-induced ovarian failure (10). Preventive measures include calcium supplementation (total intake of 1,200 mg/day), vitamin D3 (600-1,000 IU/day), and regular weight-bearing exercise (11). For those at higher risk, bisphosphonates and denosumab have been shown to prevent bone loss and reduce fracture rates (10). Recognition of the heightened and accelerated risk of bone loss in young breast cancer survivors underscores the need for early, proactive bone health management as an integral component of survivorship care.

### Cognitive dysfunction

Cognitive impairment is a prevalent problem among breast cancer survivors, even in the absence of central nervous system involvement (12). The incidence of cancer-related cognitive impairment (CRCI) in breast cancer patients can be as high as 70% during treatment and 35–60% for months to years after treatment ends (13). A recent meta-analysis estimated a pooled incidence of approximately 40%, though reported rates range widely from 9.6% to 81% depending on assessment methods used (14). Cognitive changes in young breast cancer survivors can result from chemotherapy, ET, and treatment-induced ovarian function suppression, and commonly affect executive function, memory, attention, and processing speed (15). In younger survivors, these impairments may significantly interfere with educational attainment, career progression, and productivity at a critical stage of professional development. Notably, a secondary analysis of the RxPONDER trial demonstrated that chemoendocrine therapy had a significantly greater negative impact on cognitive function compared to ET alone in both premenopausal and postmenopausal women, with cognitive scores in premenopausal patients receiving chemotherapy failing to recover to baseline even at 36 months (13). While the cognitive effects of chemotherapy are not solely mediated by ovarian function suppression, the contribution of chemotherapy-induced POI and GnRH agonist therapy to CRCI in young women remains an important area of investigation (13). Studies have found that cognitive changes frequently co-occur with depression, anxiety, fatigue, and sleep disturbance, suggesting they may be part of a symptom cluster and that neurobehavioral symptoms during chemotherapy are associated with persistent CRCI years later (16). Inflammation has been recognized as a potential contributor, with elevated cytokine levels and alterations in white matter microstructure observed in survivors with cognitive difficulties (17). Neuroimaging studies also show structural changes, including white matter damage and altered patterns of brain activity, that may result from the neurotoxic effects of chemotherapy (18). The NCCN Survivorship Guidelines recommend a stepwise approach to management, beginning with patient education, self-management strategies, and optimization of contributing factors (fatigue, depression, sleep disturbance), followed by first-line interventions including neuropsychological evaluation, cognitive rehabilitation, psychotherapy, and routine physical activity (4). A recent meta-analysis of 11 RCTs confirmed that exercise significantly improves attention, working memory, executive function, and self-reported cognitive outcomes in breast cancer survivors (19). Mindfulness-based stress reduction and cognitive training have also shown benefit, particularly for subjective cognitive complaints (20, 21) (4).The potential for chronic cognitive impairment in young breast cancer survivors highlights the need to develop preventive strategies and reinforces the importance of using biomarkers and genomic tools for treatment selection to avoid chemotherapy when it is unlikely to provide meaningful oncologic benefit (13).

### Fatigue

Cancer-related fatigue (CRF) is a common and particularly challenging symptom among cancer survivors, defined as a distressing, persistent sense of physical, emotional, and/or cognitive exhaustion that is not proportional to recent activity and is not easily relieved by rest (4). While prevalence of CRF in breast cancer patients ranges from 28% to 91%, with approximately 75% of patients experiencing increased fatigue after even a single chemotherapy cycle, the experience of fatigue in younger women is qualitatively and quantitatively distinct (22). A meta-analysis of over 12,000 breast cancer survivors found a pooled prevalence of severe fatigue of approximately 27%, with higher disease stage, chemotherapy, and the combination of surgery, radiotherapy, and chemotherapy, with or without ET, identified as significant risk factors (23). Importantly, younger age has been consistently identified as a risk factor for more severe, persistent and functionally disruptive fatigue in breast cancer survivors, with younger women reporting greater fatigue interference than older survivors (24–26). A large longitudinal study (CANTO cohort) identified that approximately 21% of breast cancer survivors belong to a high-risk fatigue trajectory with persistent severe fatigue at 4 years post-diagnosis, while an additional 19% follow a deteriorating trajectory with worsening fatigue over time; younger age, depression, insomnia, and ET were key risk factors for these unfavorable trajectories (27). In young survivors, persistent CRF may significantly impact the ability to maintain employment, care for young children, pursue educational goals, and engage in social and family roles with severe fatigue associated with reduced likelihood of employment and economic hardship (4).

The underlying causes of CRF are complex and multifactorial. Biological factors, including inflammation and immune dysregulation, have been implicated, with elevated levels of pro-inflammatory cytokines and hormonal dysregulation. The biological underpinnings of CRF in young women are closely related with treatment-induced POI. Abrupt estrogen deprivation disrupts hypothalamic-pituitary-adrenal (HPA) axis regulation and promotes chronic low-grade systemic inflammation, contributing to fatigue in young women (28, 29),. Fatigue rarely occurs in isolation and most commonly clusters with pain, emotional distress, depression, anemia, sleep disturbances, and cognitive dysfunction, suggesting shared underlying mechanisms (30). Psychosocial and behavioral factors, including pre-existing mental health conditions, socioeconomic stressors, and the emotional burden of a cancer diagnosis at a young age, may further exacerbate fatigue symptoms (24, 31). Premenopausal patients receiving chemoendocrine therapy in the RxPONDER trial failed to return to baseline fatigue scores even at 36 months, further supporting that CRF in young survivors is a chronic, biologically driven condition rather than a transient side effect (13). Its functional consequences are disproportionate in this population: severe fatigue is associated with reduced likelihood of returning to work and economic hardship, and impairs the capacity to fulfil parenting and social roles (4, 32). Despite its serious consequences, CRF remains often underdiagnosed and undertreated. Screening using patient-reported outcomes is recommended at regular intervals during and following treatment. The NCCN Guidelines recommend a stepwise, age-adapted approach to management: first, identification and treatment of contributing factors (pain, emotional distress, anemia, sleep disturbance, nutritional deficits, and comorbidities); followed by patient education and energy conservation strategies; then physical activity (category 1), which is the most strongly supported intervention (30, 33). A network meta-analysis of 77 RCTs in breast cancer patients found that cognitive behavioral therapy (CBT) was the most effective intervention for reducing CRF, followed by traditional exercises, aerobic exercise, multimodal exercise, music interventions, and yoga (34). Additional category 1 recommendations include CBT, mindfulness-based stress reduction, psycho-educational therapies, CBT for insomnia, and massage therapy. Internet-delivered CBT represents a practically important option for young women facing logistical barriers to in-person care (33). Acupuncture and bright white light therapy are also recommended as complementary approaches. Pharmacological options remain limited (4). A critical unmet need remains the absence of trials specifically enrolling young breast cancer survivors, with current recommendations largely extrapolated from mixed-age populations; future research should prioritize age-stratified analyses and address the specific contribution of treatment-induced POI to fatigue mechanisms.

### Emotional distress

Young breast cancer survivors face a disproportionate burden of emotional distress after treatment, which can take many forms, including anxiety, depression, post-traumatic stress, and fear of cancer recurrence (FCR) (35). A large pan-Canadian cohort study of women diagnosed with breast cancer at age ≤40 found that rates of clinically significant anxiety (69.1%) and depression (42.7%) were remarkably high early in diagnosis, with pre-existing depression, parenting young children (age ≤10), and low coping self-efficacy identified as key risk factors (36). A meta-analysis of over 15,000 women with breast cancer found a pooled prevalence of psychological distress of 52%, with mastectomy, advanced stage, and history of depression identified as independent risk factors (37, 38). Young breast cancer survivors may experience additional sources of distress, including altered body image, concerns about fertility and future parenthood, disruptions in intimate relationships, and work/school challenges, with adolescent and young adult (AYA) survivors significantly more likely to report emotional problems (56.7% vs 38.9%), fertility concerns (13.3% vs 0.1%), and work/school issues (13.3% vs 4.1%) compared to older survivors (39). According to the NCCN Survivorship Guidelines, “distress” in cancer survivors involves a combination of emotional, social, spiritual, and physical challenges that can interfere with coping mechanisms and daily functioning, and screening for anxiety, depression, trauma, and distress should be part of routine care (4).

FCR is the most prevalent unmet need among young breast cancer survivors, with prevalence rates as high as 70–84% in young adults (40, 41). A prospective study from the Young Women’s Breast Cancer Study found that approximately one-third of young women had FCR that was severe and did not improve or worsened over 5 years after diagnosis (42). FCR is exacerbated at key moments such as surveillance appointments and health setbacks, and is associated with reproductive concerns with 55.2% of reproductive-aged breast cancer patients belonging to a high FCR-intense profile, predicted by younger age, chemotherapy, and fertility intention (43). Post-traumatic stress is also a significant concern: approximately 6–23% of breast cancer patients report symptoms consistent with PTSD, with younger age, advanced disease, limited social support, and prior trauma history identified as key risk factors (32). This persistent distress not only affects mental well-being but also physical health, as survivors who experience untreated distress are less likely to engage in preventive health behaviors such as exercise and follow-up medical appointments (44). Without intervention, distress can lead to suicidal ideation and behavior; a meta-analysis found a pooled prevalence of suicidal ideation of 10% among women with breast cancer, with younger age identified as a significant risk factor for suicide mortality (45). Factors that increase emotional distress in young breast cancer survivors include chronic health problems, treatment-induced physical changes, low social support, financial strain, having young or dependent children, and racial disparities (46). Given these heightened risks, young breast cancer survivors should receive regular screening for anxiety, depression, trauma, and distress, with prompt referral to mental health services, preferably with psycho-oncology training, for those with elevated needs, to ensure comprehensive, age-appropriate care and improved quality of life in survivorship (44, 47).

### Sleep disorders

Sleep disorders are among the most prevalent and persistent survivorship challenges breast in young breast cancer survivors, with problems such as insomnia, excessive sleepiness, and other sleep-related movement or breathing disorders affecting a large proportion of this population. Sleep disturbance affects approximately 60% of women receiving breast cancer treatment, with poor sleep quality reported by up to 66% and clinically significant insomnia reaching 62% in breast cancer survivors, a prevalence three times higher than in the general population (48, 49). The burden is particularly pronounced in young women a longitudinal cohort of 836 premenopausal women (≤45 years) with breast cancer, 57% met criteria for clinical insomnia at diagnosis, and 42% continued to experience insomnia symptoms 3 years later (50). Factors contributing to these disorders include treatment-induced premature menopause and associated VMS (hot flashes/night sweats), which are particularly relevant in young women undergoing ovarian function suppression or chemotherapy-induced ovarian insufficiency, as well as psychological stress, chronic pain, and fatigue (51). Estrogens and progesterone independently regulate sleep-wake pathways via central mechanisms, meaning that hormonal disruption can affect sleep quality even in the absence of VMS. In multivariable analyses, hot flashes and depressive symptoms were the strongest predictors of persistent insomnia in young breast cancer survivors (50). Medication side effects, including those from ET, corticosteroids, and antiemetics, can also affect sleep quality, and survivors may adopt maladaptive habits, such as irregular sleep schedules or excessive daytime napping, that further disrupt their sleep (4). Persistent sleep problems during survivorship are often associated with ongoing symptoms such as anxiety and depression, which can exacerbate sleep disturbances in a bidirectional relationship (52). In qualitative studies, young breast cancer survivors describe difficulty falling asleep, waking at night, and not feeling refreshed, attributing interrupted sleep to VMS, anxiety, ruminating thoughts, everyday life stressors, and discomfort, with sleep disturbance persisting across 5 years of survivorship (53). These disturbances are particularly disruptive in younger women balancing work, childcare, and active lifestyles, and sleep disturbance has been associated with younger age in women undergoing adjuvant breast cancer treatment (52). Proper assessment and treatment of these sleep problems is essential. The NCCN Survivorship Guidelines recommend screening for sleep disorders at regular intervals, with assessment of contributing factors including VMS, medications, pain, fatigue, and emotional distress (4). CBT for insomnia (CBT-I) is the preferred first-line treatment, with evidence from a randomized controlled trial demonstrating that internet-delivered CBT-I produces large effect sizes for insomnia severity (Cohen’s d, 1.17) in breast cancer survivors, with improvements maintained at follow-up (54). For young survivors in whom insomnia is associated with VMS related to treatment-induced menopause, management of VMS may improve sleep quality. Because menopausal hormone therapy is generally contraindicated in women with hormone receptor-positive breast cancer, non-hormonal approaches represent the preferred management strategy, while hormone therapy may be reserved for carefully selected patients without contraindications. Sleep hygiene education should be part of a multicomponent approach but is not effective as a standalone treatment. Pharmacologic interventions may be considered as adjunctive therapy when non-pharmacologic approaches are insufficient, and referral to a sleep specialist is recommended for chronic or refractory symptoms (4). Improving sleep quality can ultimately improve the well-being and recovery outcomes of young breast cancer survivors, underscoring the need for comprehensive, age-appropriate (accounting for the specific hormonal milieu of young women on ovarian suppression) sleep management in their care.

### Weight gain

Weight gain following a breast cancer diagnosis is a common issue that disproportionately affects young survivors. Research shows that nearly 50% of breast cancer patients are overweight at the time of diagnosis (most available data derive from Western populations), with over a quarter experiencing significant weight gain within 4 years post-diagnosis (55, 56). A prospective cohort from the Young Women’s Breast Cancer Study found that one-third (33.9%) of women diagnosed at age ≤40 experienced clinically significant weight gain (≥5%) within 3 years of diagnosis (57). Premenopausal status is the strongest risk factor for weight gain, with premenopausal women having a 65% higher risk of gaining >2 kg compared to postmenopausal women (58). Weight gain in young survivors is multifactorial and may be influenced by treatment-related factors, including chemotherapy, treatment-induced menopause, and ETs such as tamoxifen (OR 2.7 for clinically significant weight gain) and aromatase inhibitors as well as reduced physical activity, metabolic changes, and psychosocial factors (59). Premenopausal women on adjuvant ET are particularly vulnerable, with a 67% cumulative incidence of clinically significant weight gain over 5 years compared to 43% in postmenopausal women (60). This weight gain is associated with heightened risks of cancer recurrence and cardiovascular disease (61). Weight gain further contribute to the deterioration of quality of life, highlighting the need for comprehensive weight management in cancer care (62–64). Notably, obesity has been identified as a significant, potentially modifiable risk factor for recurrence specifically in premenopausal women with luminal-type breast cancer (65).

Maintaining a healthy weight is therefore crucial for reducing recurrence risk and improving survival in young breast cancer survivors. Studies reveal that both being obese at diagnosis and gaining weight post-treatment are linked to poorer outcomes, including lower survival rates and increased recurrence (66). The NCCN Survivorship Guidelines recommend achieving a BMI between 18.5 and 24.9 kg/m² through caloric management, physical activity, and behavior modification (4). A Cochrane review confirmed that multimodal interventions incorporating diet, exercise, and psychosocial support resulted in greater weight reduction (−2.88 kg) and improved quality of life (67). Digital solutions offer additional support for young survivors who may be more receptive to technology-based approaches (33, 68). Despite these advancements, maintaining long-term adherence remains a challenge, indicating an ongoing need for tailored, age-appropriate weight management programs that support young breast cancer survivors in achieving optimal health outcomes.

### Vasomotor symptoms

VMS, primarily hot flashes and night sweats, are among the most prevalent and burdensome survivorship concerns in young breast cancer patients. Almost 65% of women report hot flashes during or following breast cancer treatment, with 64-82% rating their symptoms as moderate to severe (69). Six times as many breast cancer survivors experience hot flashes compared to age-matched controls, with younger age and history of ET identified as the strongest predictors of symptom activity (70). These symptoms arise from the estrogen deprivation induced by chemotherapy, ovarian suppression, and ET. Their impact extends well beyond thermoregulatory discomfort: in a cohort of 163 young breast cancer survivors, VMS were significantly associated with higher depressive symptoms, and 64% of this total effect was mediated through sleep disturbance (71). VMS can also result in early discontinuation of ET, a particularly consequential outcome given the oncological stakes of treatment adherence. Symptom management requires a multimodal approach, as systemic hormone replacement therapy is generally contraindicated in hormone receptor-positive disease (72). The NCCN Survivorship Guidelines recommend non-hormonal pharmacological options as first-line treatment, including antidepressants, anticonvulsants, and antihypertensives, and acknowledge that acupuncture, yoga, and hypnosis may also provide benefit (4). Among pharmacological agents, venlafaxine, an SNRI with minimal effect on tamoxifen metabolism, demonstrated approximately 60% reduction in hot flash frequency after four weeks and proved superior to clonidine in a randomized placebo-controlled trial. Gabapentin, oxybutynin, and low-dose paroxetine are additional evidence-based options, while fezolinetant, a novel neurokinin-3 receptor antagonist, has demonstrated VMS reductions exceeding 50% from baseline in phase III trials, though its safety profile in breast cancer patients requires further dedicated study (4, 73). On the non-pharmacological side, CBT incorporating psychoeducation, paced breathing, and cognitive restructuring has demonstrated meaningful reductions in hot flash frequency and problem rating in women treated for breast cancer, supported by randomized evidence from the MENOS trial program (74). Structured physical activity, mindfulness-based interventions, and acupuncture represent further endorsed strategies (4). Systematic assessment and proactive management of VMS should be considered a standard component of survivorship care in this population.

### Cardiovascular complications

Cardiovascular complications represent a major long-term issue in young breast cancer survivors, resulting not only from the direct vascular toxicity of anticancer therapies but also from treatment-induced POI and its metabolic consequences (75). Gonadotoxic chemotherapy and ovarian suppression strategies lead to abrupt estrogen deprivation at a younger age than natural menopause, thereby accelerating cardiovascular aging (76). Estrogen normally exerts cardioprotective effects through regulation of lipid metabolism, endothelial homeostasis, vascular tone, and adipose tissue distribution. Consequently, estrogen deficiency promotes an atherogenic lipid profile characterized by increased LDL cholesterol and triglycerides and reduced HDL cholesterol, changes that may be further amplified by aromatase inhibitors through suppression of residual peripheral estrogen synthesis (77, 78). In addition, endocrine and metabolic alterations associated with POI contribute to oxidative stress, activation of the renin–angiotensin system, insulin resistance, and chronic low-grade inflammation, all of which favor premature atherosclerosis (76). Beyond metabolic effects, vascular dysfunction is a key component of long-term cardiotoxicity. Estrogen deprivation impairs endothelial nitric oxide production and vascular relaxation, while several chemotherapeutic agents - particularly anthracyclines - independently induce endothelial injury, arterial stiffness, and an aging-like vascular phenotype (79). Clinical studies have shown reduced flow-mediated dilation, increased carotid intima-media thickness, altered left ventricular diastolic function, and higher prevalence of hypertension and metabolic syndrome in women with POI compared with healthy controls (80). Importantly, the coexistence of chemotherapy-related vascular toxicity and prolonged estrogen deprivation creates a synergistic insult that may remain clinically silent for years before manifesting as overt cardiovascular disease. Alterations in body composition further contribute to cardiometabolic risk. Loss of estrogenic regulation promotes visceral adiposity, increased free fatty acid flux to the liver, hepatic steatosis, impaired glucose metabolism, and progressive sarcopenia (76). Aromatase inhibitor therapy has additionally been associated with increased fat mass and reduced lean body mass, leading to a sarcopenic-obesity phenotype that resembles, and may even exceed, the cardiometabolic profile observed in older postmenopausal women despite occurring decades earlier (81, 82). Overall, the convergence of dyslipidemia, endothelial dysfunction, vascular stiffness, hypertension, insulin resistance, and adverse body composition substantially increases the long-term risk of ischemic heart disease and cardiovascular mortality in this population, supporting the need for dedicated cardiovascular surveillance and preventive strategies within survivorship care (80).

### Sexual concerns

Sexual dysfunction is a significant and highly prevalent problem among young breast cancer survivors, affecting quality of life and often going untreated. In a study specifically focused on women diagnosed at age ≤40, sexual dysfunction in at least one domain was reported by 68%, and high levels of reproductive concerns by 58% (83). The risk of sexual dysfunction is 3-fold higher in women diagnosed before age 50 compared to the general population (HR 3.05, 95% CI 2.65–3.51) (84). Breast cancer treatments, including chemotherapy, ET, and surgery, often result in physical changes that affect sexual function. In young women, ET is a key contributor: current ET is a significant predictor of lubrication dysfunction (OR 3.8) and vaginal discomfort (OR 8.7), while aromatase inhibitors combined with ovarian function suppression are associated with higher rates of sexual dysfunction than tamoxifen-based regimens (83). ET combined with ovarian function suppression leads to early declines in sexual functioning and enjoyment that persist at 24 months, with lack of sexual activity reported by 61% of women on ovarian suppression (85). In addition, the psychological effects of cancer, including anxiety, depression, altered body image, and concerns about fertility and future parenthood, can profoundly affect sexual health in younger women, with body image and quality of life directly correlating with sexual function scores (86). Despite the frequency of these issues, healthcare providers often avoid discussing sexual health with survivors due to limited training, discomfort, or time constraints, even though effective treatments exist (68).

For young breast cancer survivors, sexual dysfunction can manifest as decreased desire, pain, or difficulty with arousal and satisfaction. The ACS/ASCO Breast Cancer Survivorship Care Guideline reports prevalence ranges of 23-64% for decreased libido, 20-48% for arousal/lubrication concerns, 16-36% for orgasmic difficulties, and 35-38% for dyspareunia (44). Effective first-line treatments include nonhormonal vaginal lubricants and moisturizers (water-based, silicone-based, or hyaluronic acid formulations), which should be offered to all survivors reporting vaginal symptoms given their wide availability, low cost, and negligible side effects. Pelvic physiotherapy, vaginal dilators, and topical anesthetics (such as 4% lidocaine applied before penetration) can effectively address dyspareunia (68). For refractory symptoms, low-dose vaginal estrogen (rings or suppositories preferred over creams due to lower systemic absorption) may be considered after discussion of potential risks, although use of hormonal therapies in women on aromatase inhibitors is not recommended. CBT should be highly encouraged, with evidence showing improvements in sexual function, desire, arousal, and sexual distress in breast cancer survivors on ET (68). The NCCN Survivorship Guidelines recommend screening for sexual health concerns at regular intervals and referral to sexual health specialists, psychotherapy, and couples counseling as needed (4). Addressing sexual health in young breast cancer survivorship requires individualized, proactive care and open communication between healthcare providers and survivors to ensure effective and holistic management of these issues.

### Fertility and reproductive health

Fertility and reproductive health are key concerns in breast cancer survivors and should be addressed early in the disease course. The NCCN recommends that all premenopausal patients be informed about the potential impact of cancer treatments on fertility and be asked about their reproductive goals at diagnosis, with timely referral to fertility specialists when appropriate (9, 87). Established preservation strategies include oocyte and embryo cryopreservation, while ovarian tissue cryopreservation may be considered in selected cases. The use of gonadotropin-releasing hormone (GnRH) agonists during chemotherapy can be offered as an adjunct to reduce the risk of ovarian failure (4, 87).

Menstrual function is not a reliable indicator of fertility, as amenorrhea does not necessarily imply infertility and resumption of menses does not guarantee preserved reproductive capacity. Hormone-based contraception is generally discouraged, and non-hormonal methods are preferred (9). For most survivors, pregnancy after breast cancer is considered safe and not associated with increased recurrence risk or adverse fetal outcomes, although a waiting period of at least two years is commonly recommended. Temporary interruption of endocrine therapy to allow pregnancy may be considered in selected patients, although long-term safety data are still evolving (4).

Despite guideline recommendations, access to fertility preservation remains limited due to financial barriers and disparities in care (88). Importantly, fertility-related issues significantly affect quality of life, with treatment-induced amenorrhea associated with poorer sleep and sexual functioning (5, 89). Population-based data suggest that fertility preservation is associated with higher post-diagnosis childbirth rates without compromising survival (90). These findings highlight the need to integrate fertility counseling and reproductive health management into comprehensive survivorship care for young breast cancer patients.

### Social issues

The social impact of breast cancer treatment can be profound for young survivors, often changing their lives in both positive and challenging ways. While many survivors report strengthened relationships and a renewed appreciation of life, others experience ongoing distress that affects their social and practical well-being. FCR is the most prevalent concern, with prevalence rates as high as 70–84% among young adult breast cancer survivors, and approximately one-third experience severe FCR that does not improve or worsens over 5 years after diagnosis (40, 42). This fear can affect not only survivors but also their careers, and is exacerbated at key moments such as surveillance appointments and health setbacks. Employment challenges are particularly impactful for young survivors who are at a critical stage of career development: among women diagnosed under age 40, 47% of those employed at diagnosis reported increased unpaid time off, 23% reported suffering job performance, and 30% stayed at jobs primarily to maintain health insurance (91). In the CANTO cohort, 21% of breast cancer patients had not returned to work 2 years after diagnosis, with chemotherapy-trastuzumab combinations, arm morbidity, and depression significantly increasing the odds of non-return to work (92–94). Financial hardship is another significant consequence, with 47% of young breast cancer survivors experiencing financial decline due to treatment-related costs (91). A prospective cohort study from the Young Women’s Breast Cancer Study identified three distinct financial trajectories: while 55% experienced low financial difficulty, 16% had moderate to severe difficulty peaking several years after diagnosis, with Hispanic ethnicity, unemployment, and treatment-related arm symptoms as key risk factors for sustained hardship (95). Treatment-related adverse events, including lymphedema, fatigue, and cognitive dysfunction, contribute to financial toxicity through both direct costs and indirect costs such as vocational disruption (96). In summary, while cancer treatment can lead to personal growth, young breast cancer survivors face significant social and financial barriers (compounded by concerns about fertility, intimate relationships, and parenthood) that affect their overall recovery and reintegration into daily life, underscoring the need for early screening and integrated supportive care (4, 87).

## Conclusion

In summary, the journey beyond breast cancer for young survivors involves complex, ongoing challenges that extend well beyond initial treatment. As survival rates increase, so does recognition of the multifaceted needs of young breast cancer survivors, which span physical, emotional, reproductive, and social dimensions. Survivors often face chronic symptoms such as fatigue, cognitive impairment and bone density loss, as well as emotional distress, sexual dysfunction, sleep disturbance, weight gain, and social reintegration issues, all of which may be amplified by their life stage. Treatment-induced POI, fertility concerns, disruptions in career development, and challenges in forming or maintaining intimate relationships add further complexity to the survivorship experience in this population. These lasting effects underscore the need for a comprehensive, age-appropriate, patient-centered approach to survivorship care that includes routine screening, coordinated medical and psychological support, fertility preservation counseling, and proactive management of health risks. Survivorship care pathways should integrate the expertise of oncologists, primary care providers, reproductive specialists, and mental health professionals to effectively address the unique needs of young breast cancer survivors. In addition, personalized support systems and ongoing research are essential to refine interventions, minimize long-term side effects, and improve survivors’ quality of life. By prioritizing integrated, multidisciplinary care tailored to the developmental and psychosocial needs of younger women, the healthcare community can better support young breast cancer survivors and help them to not only live but thrive after cancer. This field of care continues to grow, advocating for policies and programs that focus on survivorship and recognize the importance of the well-being, resilience and long-term health of young breast cancer survivors.
